# Association of Preexisting Disability With Severe Maternal Morbidity or Mortality in Ontario, Canada

**DOI:** 10.1001/jamanetworkopen.2020.34993

**Published:** 2021-02-08

**Authors:** Hilary K. Brown, Joel G. Ray, Simon Chen, Astrid Guttmann, Susan M. Havercamp, Susan Parish, Simone N. Vigod, Lesley A. Tarasoff, Yona Lunsky

**Affiliations:** 1 Department of Health and Society, University of Toronto Scarborough, Toronto, Ontario, Canada; 2 Dalla Lana School of Public Health, University of Toronto, Toronto, Ontario, Canada; 3 Women’s College Research Institute, Women’s College Hospital, Toronto, Ontario, Canada; 4 ICES, Toronto, Ontario, Canada; 5 Li Ka Shing Knowledge Institute, St Michael’s Hospital, Toronto, Ontario, Canada; 6 Hospital for Sick Children, Toronto, Ontario, Canada; 7 Department of Pediatrics, University of Toronto, Toronto, Ontario, Canada; 8 Center for Psychiatry and Behavioral Health, Wexner Medical Center, The Ohio State University, Columbus; 9 College of Health Professions, Virginia Commonwealth University, Richmond; 10 Department of Psychiatry, University of Toronto, Toronto, Ontario, Canada; 11 Azrieli Adult Neurodevelopmental Centre, Centre for Addiction and Mental Health, Toronto, Ontario, Canada

## Abstract

**Importance:**

Severe maternal morbidity and mortality are important indicators of maternal health. Pregnancy rates are increasing in women with disabilities, but their risk of severe maternal morbidity and mortality is unknown, despite their significant social and health disparities.

**Objective:**

To determine the risk of severe maternal morbidity or mortality among women with a physical, sensory, or intellectual/developmental disability compared with women without disabilities.

**Design, Setting, and Participants:**

This population-based cohort study used linked health administrative data in Ontario, Canada, from 2003 to 2018. The cohort included all singleton births to women with preexisting physical, sensory, and intellectual/developmental disabilities as well as with 2 disabilities or more compared with women without a disability. Data analysis was conducted from September 2019 to September 2020.

**Exposures:**

Disabilities were identified with published algorithms applied to diagnoses in 2 physician visits or more or at least 1 emergency department visit or hospitalization.

**Main Outcomes and Measures:**

Severe maternal morbidity (a validated composite of 40 diagnostic and procedural indicators) or all-cause maternal mortality, arising between conception and 42 days post partum. Relative risks were adjusted for maternal age, parity, income quintile, rurality, chronic medical conditions, mental illness, and substance use disorders.

**Results:**

The cohort comprised women with physical disabilities (144 972 women; mean [SD] age, 29.8 [5.6] years), sensory disabilities (45 259 women; mean [SD] age, 29.1 [6.0] years), intellectual/developmental disabilities (2227 women; mean [SD] age, 26.1 [6.4] years), and 2 or more disabilities (8883 women; mean [SD] age, 29.1 [6.1] years), and those without disabilities (1 601 363 women; mean [SD] age, 29.6 [5.4] years). The rate of severe maternal morbidity or death was 1.7% (27 242 women) in women without a disability. Compared with these women, the risk of severe maternal morbidity or death was higher in women with a physical disability (adjusted relative risk [aRR], 1.29; 95% CI, 1.25-1.34), a sensory disability (aRR, 1.14; 95% CI, 1.06-1.21), an intellectual/developmental disability (aRR, 1.57; 95% CI, 1.23-2.01), and 2 or more disabilities (aRR, 1.74; 95% CI, 1.55-1.95). Similar aRRs were observed for severe maternal morbidity or death arising in pregnancy, from birth to 42 days post partum, and from 43 to 365 days post partum. Women with disabilities were more likely than those without disabilities to experience multiple severe maternal morbidity indicators. The most prevalent indicators in all groups were intensive care unit admission, severe postpartum hemorrhage, puerperal sepsis, and severe preeclampsia.

**Conclusions and Relevance:**

In this study, women with a preexisting disability were more likely to experience severe maternal morbidity or mortality. Preconception and perinatal care provisions should be considered among women with a disability to mitigate the risk of these rare but serious outcomes.

## Key Points

**Question:**

What is the risk of severe maternal morbidity or mortality in women with a preexisting disability compared with women without disabilities?

**Findings:**

This population-based cohort study evaluated more than 1.8 million births in Ontario, Canada. Compared with women without a disability, the adjusted relative risk of severe maternal morbidity or death was 29% higher among women with a physical disability, 14% higher among women with a sensory disability, 57% higher among women with an intellectual/developmental disability, and 74% higher among women with 2 or more disabilities.

**Meaning:**

These findings suggest that preconception and perinatal care provisions should be considered among women with a disability to reduce the risk of adverse outcomes.

## Teaser

This cohort study assesses the risk of severe maternal morbidity or mortality among women with a physical, sensory, or intellectual/developmental disability compared with women without disabilities.

## Introduction

Severe maternal morbidity and mortality are important indicators of maternal health. Maternal mortality, defined as death in pregnancy or at less than 42 days after giving birth, can be a direct result of obstetric complications or interventions or an indirect result of preexisting or new disease aggravated by pregnancy.^1,2^ Although maternal mortality is rare in industrialized countries, rates since 1990 have increased in the United States (from 10.0 to 17.3 deaths per 100 000 births)^2^ and Canada (7.6 to 11.0 deaths per 100 000 births).^1^ Severe maternal morbidity is defined by disease-specific criteria, such as eclampsia; interventions, such as blood transfusion; or organ system dysfunction, such as heart failure, each on the continuum to mortality.^3^ Similar to maternal mortality, severe maternal morbidity rates increased in Canada by 1.3% per year in 2004 to 2015 to 17.7 per 1000 births,^4^ with even greater increases in the United States.^5^ Severe maternal morbidity and mortality have devastating effects on families^6^ and are largely preventable. Studies showing that chronic conditions, such as cardiovascular disease and diabetes, are among the leading causes of such morbidity and mortality have led to significant preventive efforts for women with these conditions, including better preconception and perinatal care.^7^ However, the association of maternal disability with these outcomes is relatively unknown.

Disability is common in women of reproductive age, with a prevalence of at least 12% in those aged 15 to 49 years.^8,9^ Disabilities are heterogeneous and can be present since birth or acquired, be continuous or episodic, and limit functioning or life expectancy. Broadly classified, physical disabilities affect mobility, flexibility, or dexterity; sensory disabilities affect hearing or vision; and intellectual/developmental disabilities affect cognitive and conceptual, social, or practical skills. During the last 20 years, pregnancy rates in women with disabilities have increased.^10,11^ Accumulating evidence suggests that, compared with those without disabilities, women with disability have elevated risk of pregnancy complications, such as gestational hypertension.^12^ However, few studies have examined the association of disability with severe maternal morbidity,^13,14,15^ and only 1 small clinical study measured maternal mortality.^13^ This is an important omission because women with disabilities experience social and health disparities known to increase risks of severe maternal morbidity and mortality^16,17,18,19,20^ and could benefit from preconception and perinatal efforts to prevent these outcomes. In a population-based cohort in Ontario, Canada, our objective was to determine the risk of severe maternal morbidity or mortality in women with physical, sensory, intellectual/developmental, and multiple disabilities compared with women without any such disability.

## Methods

### Study Design and Setting

We conducted a population-based cohort study in Ontario, Canada, following the Strengthening the Reporting of Observational Studies in Epidemiology (STROBE) reporting guideline.^21^ Ontario is the largest province in Canada, with 14.7 million residents and 140 000 births yearly. Under a universal health care system, all medically necessary physician and hospital services, including prenatal and postpartum medical care, are delivered to Ontario residents at no direct cost. We included women aged 15 to 49 years with and without disabilities who had a singleton live birth or stillbirth conceived between April 1, 2003, and March 31, 2018, and who were eligible for Ontario’s health insurance plan in the 2 years before cohort entry. Women were followed up from conception to 365 days post partum to ascertain outcomes, to a maximum of December 31, 2019.

Data were obtained from ICES, a prescribed entity under section 45 of Ontario’s Personal Health Information Protection Act. Section 45 authorizes ICES to collect personal health information, without consent, for the purpose of analysis or compiling statistical information with respect to the management of, evaluation or monitoring of, the allocation of resources to, or planning for all or part of the health system. Projects conducted under section 45, by definition, do not require review by a research ethics board. This project was conducted under section 45, and approved by ICES’s Privacy and Legal Office.

### Data Sources

We accessed health administrative data at ICES (Toronto, Ontario), an independent, not-for-profit organization that houses diagnostic, procedural, and sociodemographic data for Ontario residents. We captured births in the MOMBABY data set, which holds records for approximately 98% of Ontario births. These data were linked deterministically with hospitalizations, emergency department visits, physician visits, census data, and vital statistics with a unique encoded identifier and analyzed at ICES (eTable 1 in the Supplement). Primary hospital diagnoses, physician billing claims, and sociodemographic data have been shown to be complete and accurate.^22^

### Exposure

Our definitions of physical, sensory, and intellectual/developmental disabilities were derived from algorithms developed to measure disability in administrative data.^23,24^ From these algorithms, we developed an initial list of codes, to which we added codes if they were judged by a group of 13 clinicians with disability expertise to be likely to result in functional limitations, as defined by the *International Classification of Functioning, Disability and Health*^26^; and were classified as being chronic by the Agency for Healthcare Research and Quality *Chronic Condition Indicator *(*CCI*)* for International Statistical Classification of Diseases, Tenth Revision, Clinical Modification *(*ICD-10-CM*).^25^ A disability was said to be present if a relevant diagnosis was recorded in 2 physician visits or more or at least 1 emergency department visit or hospitalization from database inception to conception, the latter estimated by subtracting gestational age from the birth date.^27^ Physical disabilities were congenital anomalies, musculoskeletal or neurologic disorders, and permanent injuries; sensory disabilities were hearing and vision loss; and intellectual/developmental disabilities were autism spectrum disorder, chromosomal anomalies resulting in intellectual disability, fetal alcohol spectrum disorder, and intellectual disability (eTable 2 in the Supplement). Women with diagnoses in at least 2 of these categories (ie, with multiple disabilities) were categorized separately. The referent group was women without any recognized disability.

### Outcomes

Our primary outcome was a composite of severe maternal morbidity or mortality. The former was measured with a definition developed and validated by the Canadian Perinatal Surveillance System^3^ and adapted^28^ to include 40 diagnostic (eg, eclampsia) and procedural indicators (eg, peripartum hysterectomy) arising in pregnancy or up to 42 days post partum (eTable 3 in the Supplement). Maternal mortality was defined as death from any cause around the time of birth or within 42 days post partum.^6^ As secondary outcomes, we assessed severe maternal morbidity or mortality within pregnancy, birth to 42 days post partum, and 43 to 365 days post partum separately, and maternal mortality and specific severe maternal morbidity indicators alone, each measured from conception to 365 days post partum.

### Covariates

Covariates were maternal age, parity, and social and health disparities known to affect women with disabilities.^16,17,18,19,20^ Neighborhood income quintile was measured by linking census area–level income data with residential postal codes. Rural residence was measured with the Rurality Index of Ontario,^29^ which uses 10 indicators, such as travel time to different levels of care, to classify neighborhoods as rural (≥45 points) or urban (≤44 points). We used validated disease registries to capture diabetes,^30^ chronic hypertension,^31^ and cardiovascular disease^32,33,34,35^ diagnosed before conception. We used the Johns Hopkins Adjusted Clinical Groups system version 10.0 collapsed ambulatory diagnostic groups (excluding disability diagnoses) to identify stable and unstable comorbid chronic conditions more broadly.^36^ We measured any mental illness and substance use disorders on the basis of 2 or more physician visits or at least 1 emergency department visit or hospitalization less than 2 years before conception.^37,38^ We also measured the occurrence of a first-trimester prenatal care visit and the total number of visits.

### Statistical Analysis

We described the baseline characteristics of women with physical, sensory, intellectual/developmental, and multiple disabilities by using frequencies and proportions, and compared these with those of women without disabilities, using standardized differences. Standardized differences use effect size methods to identify meaningful differences between groups that, unlike *P* values, are not influenced by sample size. Values greater than 0.10 are clinically significant.^39^ We used modified Poisson regression to directly estimate relative risks and 95% CIs for the binary outcome of severe maternal morbidity or mortality between conception and 42 days post partum in each disability group vs women with no disability,^40^ with a robust variance estimator to account for multiple births to the same mother.^41^ We conducted 2 sets of multivariable models, the first minimally adjusted for age, parity, neighborhood income quintile, and rurality, and the second fully adjusted for these sociodemographic variables and health variables that could plausibly be on the causal pathway between disability status and the outcome (ie, type 1 or 2 diabetes, chronic hypertension or cardiovascular disease, stable and unstable chronic conditions, mental illness, and substance use disorders). Analyses were also conducted for severe maternal morbidity or mortality in pregnancy, birth to 42 days post partum, and 43 to 365 days post partum separately as well as maternal mortality from birth to 365 days post partum. We used cumulative logistic regression to compare the odds of 1 or more (vs 0), 2 or more (vs ≤1), and 3 or more (vs ≤2) severe maternal morbidity indicators in each disability group vs women with no disability. Finally, we used frequencies and proportions to describe the 3 most prevalent severe maternal morbidity indicators in each group.

We undertook several additional analyses. First, the disability algorithms included a range of conditions whose characteristics, including age at diagnosis and proximity of disability-related health care use to conception, could affect health in pregnancy. We described outcome rates by age at diagnosis (ie, first recorded at <15 years vs later) because disabilities diagnosed in childhood could carry greater stigma and result in greater social and health disparities than those acquired later. We also described outcome rates by proximity of disability-related health care use to conception (ie, recorded <1 year before conception vs only earlier) because disability-related health care use closer to conception could indicate more active symptoms near pregnancy than only distal encounters. Second, we examined severe maternal morbidity or mortality between conception and 365 days post partum by disability subtype to explore heterogeneity in risk. Third, we stratified models examining severe maternal morbidity or mortality between birth and 365 days post partum by delivery mode (ie, cesarean or vaginal) and birth outcome (ie, live birth or stillbirth) because these affect risk.^42,43^ Analyses used SAS version 9.4 (SAS Institute Inc).

## Results

There were 144 972 births for women with a physical disability, 45 259 for those with a sensory disability, 2227 for those with an intellectual/developmental disability, 8883 for those with multiple disabilities, and 1 601 363 for those without a disability. Compared with women without a disability (mean [SD] age, 29.6 [5.4] years), those with sensory disabilities (mean [SD] age 29.1 [6.0] years), intellectual/developmental disabilities (mean [SD] age, 26.1 [6.4] years), and multiple disabilities (mean [SD] age, 29.1 [6.1] years) were younger and those with physical disabilities were slightly older (mean [SD] age 29.8 [5.6] years) (Table 1). Women with intellectual/developmental disabilities were more likely to live in low-income neighborhoods. Women with sensory and multiple disabilities were more likely to have diabetes, and those with multiple disabilities were more likely to have chronic hypertension or cardiovascular disease. Women with physical and multiple disabilities were more likely to have stable chronic conditions, and all were more likely to have unstable chronic conditions. All were more likely to have mental illness, and those with intellectual/developmental and multiple disabilities were more likely to have a substance use disorder (Table 1).

**Table 1.** Baseline Characteristics of Women With a Disability vs Those Without a Disability Who Had a Singleton Birth in Ontario, Canada, 2003-2018

Variable	Physical disability only (n = 144 972)	Sensory disability only (n = 45 259)	Intellectual/developmental disability only (n = 2227)	Multiple disabilities (n = 8883)	No disability (n = 1 601 363)
Age, y					
Mean (SD)	29.78 (5.60)	29.06 (5.95)	26.14 (6.43)^a^	29.07 (6.05)	29.56 (5.40)
15-24	25 980 (17.9)	10 561 (23.3)^a^	1006 (45.2)^a^	2178 (24.5)^a^	283 117 (17.7)
25-34	89 479 (61.7)	26 138 (57.8)^a^	951 (42.7)^a^	4935 (55.6)^a^	1 029 218 (64.3)
35-49	29 513 (20.4)	8550 (18.9)	270 (12.1)^a^	1770 (19.9)	289 028 (18.0)
Multiparous	84 392 (58.2)	24 600 (54.4)	1255 (56.4)	4962 (55.9)	914 219 (57.1)
Neighborhood income quintile					
1, lowest	31 023 (21.4)	10 051 (22.2)	837 (37.6)^a^	2303 (25.9)	353 505 (22.1)
2	28 884 (19.9)	9173 (20.3)	485 (21.8)	1798 (20.2)	322 413 (20.1)
3	29 658 (20.5)	9243 (20.4)	398 (17.9)	1755 (19.8)	327 804 (20.5)
4	30 566 (21.1)	9369 (20.7)	266 (11.9)^a^	1685 (19.0)	329 660 (20.6)
5, highest	24 226 (16.7)	7274 (16.1)	224 (10.1)^a^	1310 (14.7)	261 877 (16.4)
Missing	615 (0.4)	139 (0.3)	17 (0.8)	32 (0.4)	6104 (0.4)
Region of residence					
Rural	8281 (5.7)	2014 (4.5)	113 (5.1)	499 (5.6)	67 026 (4.2)
Missing	2192 (1.5)	518 (1.1)	59 (2.6)^a^	126 (1.4)	19 891 (1.2)
Type 1 or 2 diabetes	3663 (2.5)	1367 (3.0)^a^	62 (2.8)	463 (5.2)^a^	24 466 (1.5)
Chronic hypertension or cardiovascular disease	5496 (3.8)	1415 (3.1)	38 (1.7)	489 (5.5)^a^	36 865 (2.3)
Stable chronic conditions	39 998 (27.6)^a^	11 739 (25.9)	555 (24.9)	2877 (32.4)^a^	367 257 (22.9)
Unstable chronic conditions	23 524 (16.2)^a^	6604 (14.6)^a^	338 (15.2)^a^	2003 (22.5)^a^	180 985 (11.3)
Mental illness	28 488 (19.7)^a^	7841 (17.3)^a^	837 (37.6)^a^	2378 (26.8)^a^	202 064 (12.6)
Substance use disorder	2848 (2.0)	548 (1.2)	141 (6.3)^a^	302 (3.4)^a^	14 302 (0.9)
First-trimester prenatal care visit	131 749 (90.9)	40 881 (90.3)	1871 (84.0)^a^	8032 (90.4)	1 438 982 (89.9)
Total prenatal care visits, median (IQR). No.	15 (11-18)	15 (11-18)	14 (10-18)	15 (12-19)^a^	14 (11-17)

Abbreviation: IQR, interquartile range.

^a^ Standardized difference greater than 0.10 compared with women without a disability.

Risk of severe maternal morbidity or mortality between conception and 42 days post partum was higher in women with physical disabilities (3444 women [2.4%]), sensory disabilities (931 women [2.1%]), intellectual/developmental disabilities (67 women [3.0%]), and multiple disabilities (314 women [3.5%]) compared with women without them (27 242 women [1.7%]). In fully adjusted models, risks remained elevated in women with physical disabilities (adjusted relative risk [aRR], 1.29; 95% CI, 1.25-1.34), sensory disabilities (aRR, 1.14; 95% CI, 1.06-1.21), intellectual/developmental disabilities (aRR, 1.57; 95% CI, 1.23-2.01), and multiple disabilities (aRR, 1.74; 95% CI, 1.55-1.95) compared with women with no disabilities (Table 2). Although maternal mortality was too rare to report for each disability group separately, there were 13.9 deaths per 100 000 births in women with disabilities as a whole compared with 7.7 per 100 000 in women without disabilities (minimally aRR, 1.77; 95% CI, 1.17-2.69; fully aRR, 1.48; 95% CI, 0.97-2.26).

**Table 2.** Risk of Severe Maternal Morbidity or Mortality Arising Between Conception and up to 42 Days Post Partum in Women With a Disability Compared With Women Without a Disability

Variable	Individuals with outcome, No. (%)	RR (95% CI)		
		Unadjusted	Model 1^a^	Model 2^b^
Disability type				
None (n = 1 601 363)	27 242 (1.7)	1 [Reference]	1 [Reference]	1 [Reference]
Physical only (n = 144 972)	3444 (2.4)	1.39 (1.34-1.44)	1.38 (1.33-1.43)	1.29 (1.25-1.34)
Sensory only (n = 45 249)	931 (2.1)	1.21 (1.13-1.29)	1.20 (1.12-1.28)	1.14 (1.06-1.21)
Intellectual/developmental only (n = 2227)	67 (3.0)	1.70 (1.33-2.18)	1.73 (1.35-2.21)	1.57 (1.23-2.01)
Multiple (n = 8883)	314 (3.5)	2.09 (1.86-2.34)	2.05 (1.83-2.30)	1.74 (1.55-1.95)
Age, y				
15-24 (n = 322 842)	5687 (1.8)	1.05 (1.02-1.09)	0.92 (0.90-0.95)	0.96 (0.93-0.99)
25-34 (n = 1 150 721)	18 777 (1.6)	1 [Reference]	1 [Reference]	1 [Reference]
35-49 (n = 329 131)	7534 (2.3)	1.41 (1.37-1.44)	1.51 (1.47-1.55)	1.43 (1.39-1.47)
Parity				
Primiparous (n = 773 266)	16 380 (2.1)	1 [Reference]	1 [Reference]	1 [Reference]
Multiparous (n = 1 029 428)	15 618 (1.5)	0.72 (0.70-0.73)	0.67 (0.65-0.68)	0.65 (0.64-0.67)
Neighborhood income quintile				
1, lowest (n = 397 719)	7985 (2.0)	1.19 (1.15-1.24)	1.26 (1.22-1.31)	1.24 (1.19-1.28)
2 (n = 362 753)	6335 (1.7)	1.06 (1.02-1.10)	1.10 (1.06-1.14)	1.08 (1.04-1.12)
3 (n = 368 858)	6363 (1.7)	1.05 (1.01-1.09)	1.08 (1.04-1.12)	1.07 (1.03-1.11)
4 (n = 371 546)	6264 (1.7)	1.02 (0.98-1.06)	1.04 (1.00-1.08)	1.04 (1.00-1.07)
5, highest (n = 294 911)	4866 (1.6)	1 [Reference]	1 [Reference]	1 [Reference]
Region of residence				
Urban (n = 1 701 975)	29 752 (1.7)	1 [Reference]	1 [Reference]	1 [Reference]
Rural (n = 77 933)	1557 (2.0)	1.14 (1.09-1.20)	1.19 (1.13-1.25)	1.21 (1.15-1.28)
Type 1 or 2 diabetes				
Absent (n = 1 772 673)	30 838 (1.7)	1 [Reference]	NA	1 [Reference]
Present (n = 30 021)	1160 (3.9)	2.21 (2.08-2.34)	NA	1.61 (1.51-1.71)
Chronic hypertension or cardiovascular disease				
Absent (n = 1 758 391)	30 247 (1.7)	1 [Reference]	NA	1 [Reference]
Present (n = 44 303)	1751 (4.0)	2.31 (2.20-2.43)	NA	1.79 (1.70-1.88)
Stable chronic conditions				
Absent (n = 1 380 268)	22 418 (1.6)	1 [Reference]	NA	1 [Reference]
Present (n = 422 426)	9580 (2.3)	1.39 (1.36-1.42)	NA	1.20 (1.17-1.23)
Unstable chronic conditions				
Absent (n = 1 589 240)	26 322 (1.7)	1 [Reference]	NA	1 [Reference]
Present (n = 213 454)	5676 (2.7)	1.59 (1.54-1.64)	NA	1.43 (1.39-1.47)
Mental illness				
Absent (n = 1 561 086)	26 728 (1.7)	1 [Reference]	NA	1 [Reference]
Present (n = 241 608)	5270 (2.2)	1.27 (1.23-1.31)	NA	1.18 (1.14-1.22)
Substance use disorder				
Absent (n = 1 784 553)	31 508 (1.8)	1 [Reference]	NA	1 [Reference]
Present (n = 18 141)	490 (2.7)	1.45 (1.32-1.59)	NA	1.30 (1.18-1.43)

Abbreviation: NA, not applicable; RR, relative risk.

^a^ Adjusted for maternal age, parity, neighborhood income quintile, and region of residence.

^b^ Adjusted for maternal age, parity, neighborhood income quintile, region of residence, type 1 or 2 diabetes, chronic hypertension or cardiovascular disease, stable and unstable chronic conditions, mental illness, and substance use disorders.

**Figure 1.**
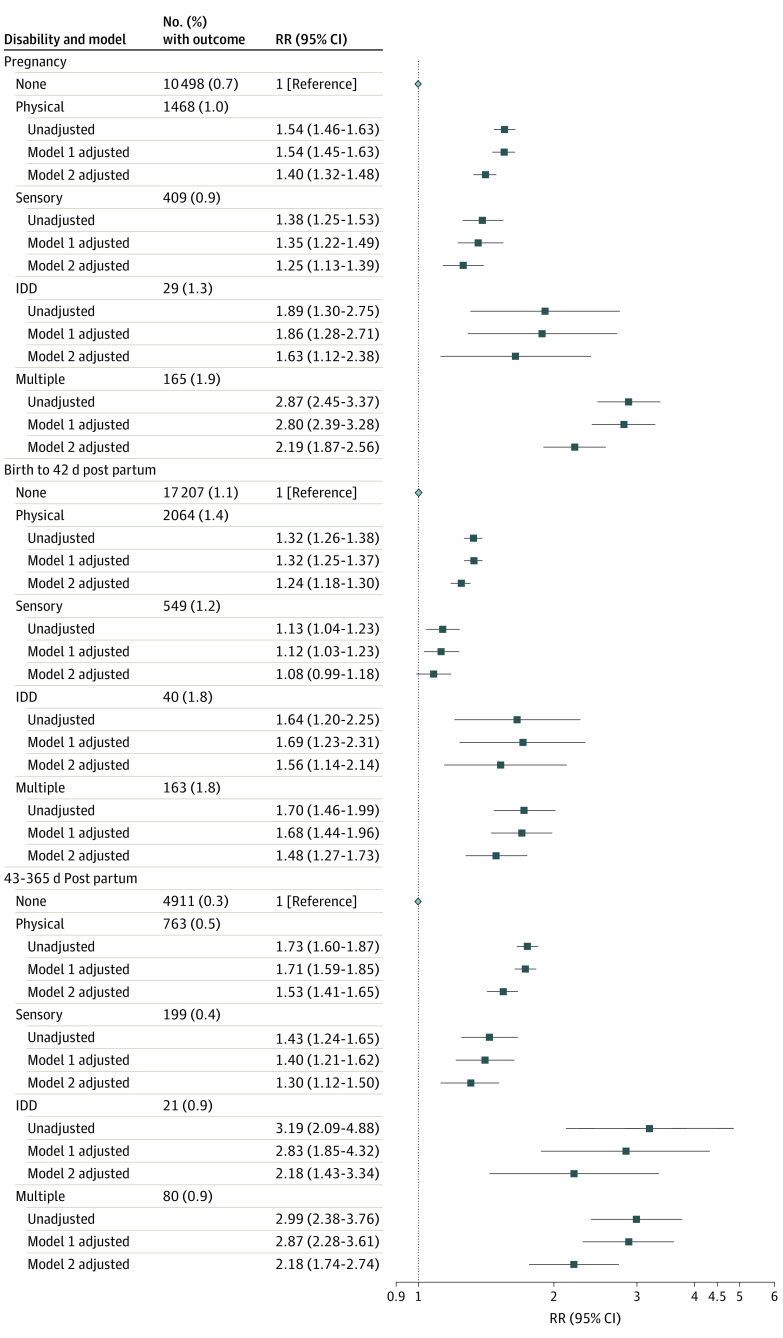
Risk of Severe Maternal Morbidity or Mortality, by Timing, in Women With a Disability Compared With Women Without a Disability Model 1 was adjusted for maternal age, parity, neighborhood income quintile, and region of residence. Model 2 was adjusted for maternal age, parity, neighborhood income quintile, region of residence, type 1 or 2 diabetes, chronic hypertension or cardiovascular disease, stable and unstable chronic conditions, mental illness, and substance use disorders. IDD indicates intellectual/developmental disability; RR, relative risk.

**Figure 2.**
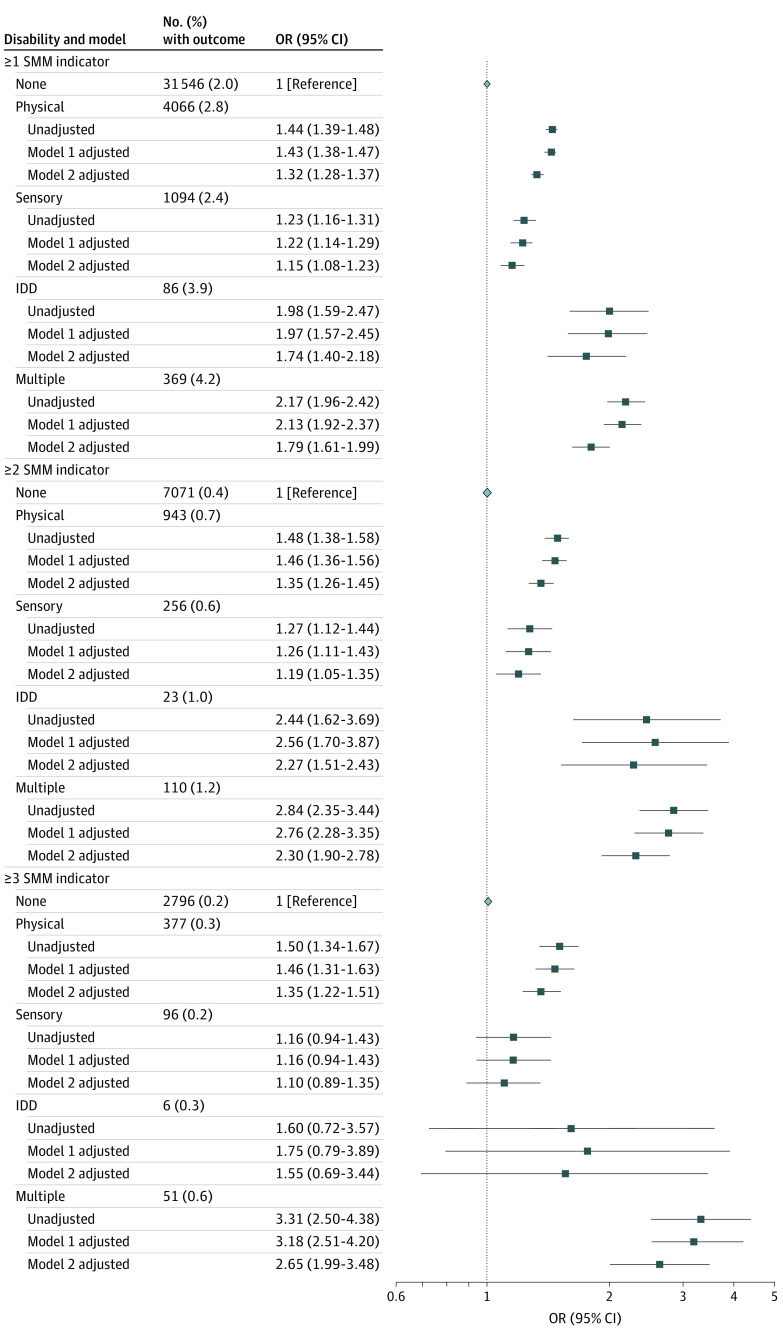
Risk of Having a Higher Number of Indicators of Severe Maternal Morbidity (SMM) Arising Between Conception and 365 Days Post Partum in Women With a Disability Compared With Women Without a Disability Model 1 was adjusted for maternal age, parity, neighborhood income quintile, and region of residence. Model 2 was adjusted for maternal age, parity, neighborhood income quintile, region of residence, type 1 or 2 diabetes, chronic hypertension or cardiovascular disease, stable and unstable chronic conditions, mental illness, and substance use disorders. IDD indicates intellectual/developmental disability; OR, odds ratio; SMM, severe maternal morbidity.

Risks of severe maternal morbidity or mortality were consistently elevated among women in each disability group compared with women without disabilities within pregnancy, birth to 42 days post partum, and 43 to 365 days post partum (fully aRRs, 1.08 [95% CI, 0.99-1.18] to 2.19 [95% CI, 1.87-2.56]) (Figure 1). Women with disabilities also had increased odds of 1 or more severe maternal morbidity indicators, 2 or more indicators, and 3 or more indicators compared with women without disabilities (Figure 2). Types of severe maternal morbidity were similar across the 5 groups, with the 3 most prevalent severe maternal morbidity indicators being a combination of severe postpartum hemorrhage; intensive care unit admission; puerperal sepsis; or severe preeclampsia and hemolysis, elevated liver enzymes, and low platelet count syndrome (Table 3).

**Table 3.** Three Most Prevalent Indicators of Severe Maternal Morbidity in Women With a Disability Compared With Women Without a Disability

Disability status	First most prevalent		Second most prevalent		Third most prevalent	
	SMM indicator	No. (%)^a^	SMM indicator	No. (%)^a^	SMM indicator	No. (%)^a^
Physical only (n = 3436 with SMM)	Postpartum hemorrhage with RBC transfusion, procedures to the uterus or hysterectomy	789 (23.0)	Maternal ICU admission	756 (22.0)	Puerperal sepsis	512 (14.9)
Sensory only (n = 931 with SMM)	Postpartum hemorrhage with RBC transfusion, procedures to the uterus or hysterectomy	218 (23.4)	Maternal ICU admission	185 (19.9)	Severe preeclampsia and HELLP syndrome	134 (14.4)
Intellectual/developmental only (n = 67 with SMM)	Maternal ICU admission	22 (32.8)	Postpartum hemorrhage with RBC transfusion, procedures to the uterus or hysterectomy	17 (25.4)	Severe preeclampsia and HELLP syndrome	7 (10.4)
Multiple (n = 314 with SMM)	Maternal ICU admission	93 (29.6)	Postpartum hemorrhage with RBC transfusion, procedures to the uterus or hysterectomy	69 (22.0)	Puerperal sepsis	39 (12.4)
None (n = 27 205 with SMM)	Postpartum hemorrhage with RBC transfusion, procedures to the uterus or hysterectomy	6903 (25.4)	Maternal ICU admission	5135 (18.9)	Puerperal sepsis	4223 (15.5)

Abbreviations: HELLP, hemolysis, elevated liver enzymes, and low platelet count; ICU, intensive care unit; RBC, red blood cell; SMM, severe maternal morbidity.

^a^ Refers to individuals with SMM who had the indicator. A given birth may have more than 1 SMM indicator.

Among women with disabilities, risks of severe maternal morbidity or mortality were generally higher in women with a first diagnosis at younger than 15 years vs later and in women with a disability-related health care encounter recorded less than 1 year before conception vs only earlier (eTable 4 in the Supplement). Risks were consistently elevated by subtype of disability, with greatest risk in women with cooccurring physical, sensory, and intellectual/developmental disabilities (aRR, 3.10; 95% CI, 1.60-6.01) (eTable 5 in the Supplement). When analyses were stratified by delivery mode, risks were elevated in all disability groups compared with women without disabilities for cesarean births and in all disability groups except women with sensory disabilities for vaginal births (eTable 6 in the Supplement). When analyses were stratified by birth outcome, risks were elevated in all disability groups for live births. Risks were also elevated for stillbirths but were not significant, likely owing to small numbers (eTable 6 in the Supplement).

## Discussion

This large population-based study demonstrated that women with physical, sensory, and intellectual/developmental disabilities were at increased risk of life-threatening complications or death in pregnancy and within 6 weeks of childbirth. These risks extended beyond the immediate postpartum period to the entire first year post partum. Women with disabilities were also at elevated risk of experiencing multiple severe maternal morbidity indicators, and their most prevalent complications were similar to those of women without disabilities. Severe maternal morbidity and mortality have devastating effects on families. Our results have implications for development of supports to assist women with disabilities, including improved preconception care to address social and health risks and comprehensive care across pregnancy and the extended postpartum period.

Few studies have examined the association between disability and severe maternal morbidity or mortality. Three studies examined severe maternal morbidity indicators in specific disability groups.^13,14,15^ In a US clinical study with 68 participants, Morton et al^13^ identified 3 cases of uterine hemorrhage and no placental abruptions in women with physical disabilities. In a US retrospective cohort study with 7098 participants, Schiff et al^14^ did not find elevated risk for placental abruption or placenta previa among Deaf women. In a retrospective cohort study in Ontario with 386 706 participants, Brown et al^15^ found that women with intellectual/developmental disabilities were at elevated risk for severe obstetric morbidity (eg, placental abruption) and systemic complications (eg, myocardial infarction). To our knowledge, there have been no population-based studies of maternal mortality in women with disabilities.^12^ Morton et al^13^ examined maternal mortality but found no deaths in their small sample. Our study adds to an almost absent literature on severe maternal morbidity and mortality in women with disabilities by leveraging large, population-based data sets needed to examine these rare outcomes. Our data also show that risk of severe maternal morbidity or mortality is elevated in women with disabilities even in a universal health care system in which health insurance is not a barrier to care.

Our study provides insight into factors that may lead to elevated risk of severe maternal morbidity or mortality among women with disabilities. Several important contributors to severe maternal morbidity and mortality, such as diabetes, cardiovascular disease,^7^ and poverty,^44^ occur more frequently in women with disabilities than in those without disabilities.^16,17,18,19,20^ In our study, risks for severe maternal morbidity or mortality were attenuated after controlling for these and other disparities. However, even after adjustment, risks remained elevated. It is possible other unmeasured factors explain some of this remaining risk. For example, disability prevalence is disproportionately high among racial/ethnic minorities,^45^ and there are well-established racial/ethnic disparities in severe maternal morbidity and mortality risk.^46^ However, Ontario health records do not collect race/ethnicity data. Similarly, unmeasured systemic issues such as gaps in the quality of perinatal care could explain our results. Future research should examine their influence on the observed associations.

Our findings have implications for preconception and perinatal care of women with disabilities. Women with chronic conditions, such as diabetes and cardiovascular disease, receive enhanced preconception care to improve disease management before pregnancy and reduce risks of severe maternal morbidity and mortality.^47^ Our data suggest women with disabilities could benefit from such supports, given their elevated risk of severe maternal morbidity and mortality, which was partly explained by cooccurring chronic conditions. Preconception care for women with disabilities should also consider how disability shapes health care access^16,20^ and interacts with other factors such as poverty.^18^ Our data demonstrate that women with disabilities require more than just proactive preconception care. Elevated risk of severe maternal morbidity or mortality extended across the first postpartum year. Most North American women receive only 1 routine postpartum health care visit at 6 weeks. Comprehensive care for women with disabilities is required well beyond this time. Women with disabilities may benefit from more frequent and longer visits across pregnancy and the first year post partum to facilitate enhanced screening and follow-up.

### Limitations

This study has limitations. Disability status may have been misclassified if a health care professional did not record a disability diagnosis in the medical chart, if a woman with a disability did not seek health care for her disability, or if she received a diagnosis before migrating to Ontario without further record of the fact thereafter. Use of administrative data to identify disability reflects a medical model of disability and does not capture the heterogeneity of activity limitations and participation restrictions experienced by women with disabilities nor the societal barriers that affect these experiences.^48^ Although we used validated measures of severe maternal morbidity and mortality,^28^ different jurisdictions use definitions that vary by included indicators and timing. Findings may not be generalizable to all settings.^49^ Furthermore, we could not determine the exact timing of a severe maternal morbidity or fatality event during the birth hospitalization, such as in relation to the timing of the birth. Hence, we may have classified a small number of events as arising post partum when they may have actually occurred antepartum or intrapartum. Although we controlled for an area-level indicator of income, we had no individual-level socioeconomic data, which may have masked some of the differences between women with and without disabilities. Although there are important racial/ethnic disparities in both the prevalence of life-threatening pregnancy complications and the burden of disability,^45,46^ we could not account for the role of racism in our findings.^50^ Quality of health care or presence of other supports, also unmeasured, could explain some of the observed associations.

## Conclusions

In this study, women with disabilities were at elevated risk for severe maternal morbidity and mortality. These findings demonstrate an urgent need to improve preconception and perinatal supports for women with disabilities to prevent these rare but devastating outcomes.

## Notes

Supplement. **eTable 1. **Details of Health Administrative Data Sources **eTable 2.** Details of How a Physical, Sensory, or Intellectual/Developmental Disability Was Determined **eTable 3.** Details of How Severe Maternal Morbidity and Mortality Were Determined **eTable 4.** Risk of Severe Maternal Morbidity or Mortality Arising Between Conception and 365 Days Post Partum in Women With a Disability by (a) the Age at Which a Disability Was Diagnosed and (b) the Timing of a Disability-Related Health Care Encounter Prior to Conception Compared With Women Without a Disability **eTable 5.** Risk of Severe Maternal Morbidity or Mortality Arising Between Conception and 365 Days Post Partum in Women With a Disability, by Subtype of Disability, Compared With Women Without a Disability **eTable 6.** Risk of Severe Maternal Morbidity or Mortality, Arising Between Birth and 365 Days Post Partum in Women With a Disability, Compared With Women Without a Disability, Stratified by (a) Delivery Mode and (b) Birth Outcome

## References

[1] Verstraeten BS, Mijovic-Kondejewski J, Takeda J, Tanaka S, Olson DM Canada’s pregnancy-related mortality rates: doing well but room for improvement. Clin Invest Med. 2015;38(1):E15-E22. doi:10.25011/cim.v38i1.22410 25662620

[2] Hirshberg A, Srinivas SK Epidemiology of maternal morbidity and mortality. Semin Perinatol. 2017;41(6):332-337. doi:10.1053/j.semperi.2017.07.007 28823579

[3] Joseph KS, Liu S, Rouleau J, Severe maternal morbidity in Canada, 2003 to 2007: surveillance using routine hospitalization data and *ICD-10CA* codes. J Obstet Gynaecol Can. 2010;32(9):837-846. doi:10.1016/S1701-2163(16)34655-2 21050516

[4] Aoyama K, Ray JG, Pinto R, Temporal variations in incidence and outcomes of critical illness among pregnant and postpartum women in Canada: a population-based observational study. J Obstet Gynaecol Can. 2019;41(5):631-640. doi:10.1016/j.jogc.2018.07.021 30385209

[5] Callaghan WM, Creanga AA, Kuklina EV Severe maternal morbidity among delivery and postpartum hospitalizations in the United States. Obstet Gynecol. 2012;120(5):1029-1036. doi:10.1097/AOG.0b013e31826d60c5 23090519

[6] Creanga AA, Berg CJ, Ko JY, Maternal mortality and morbidity in the United States: where are we now? J Womens Health (Larchmt). 2014;23(1):3-9. doi:10.1089/jwh.2013.4617 24383493 PMC3880915

[7] Kuklina E, Callaghan W Chronic heart disease and severe obstetric morbidity among hospitalisations for pregnancy in the USA: 1995-2006. BJOG. 2011;118(3):345-352. doi:10.1111/j.1471-0528.2010.02743.x 21091604

[8] Horner-Johnson W, Moe EL, Stoner RC, Contraceptive knowledge and use among women with intellectual, physical, or sensory disabilities: a systematic review. Disabil Health J. 2019;12(2):139-154. doi:10.1016/j.dhjo.2018.11.006 30473221 PMC13719107

[9] Statistics Canada Canadian Survey on Disability: Data Tables. Statistics Canada; 2013. Catalogue no. 89 654X-No. 001.

[10] Horner-Johnson W, Biel FM, Darney BG, Caughey AB Time trends in births and cesarean deliveries among women with disabilities. Disabil Health J. 2017;10(3):376-381. doi:10.1016/j.dhjo.2017.02.009 28431988 PMC5544013

[11] Brown HK, Chen S, Guttmann A, Rates of recognized pregnancy in women with disabilities in Ontario, Canada. Am J Obstet Gynecol. 2020;222(2):189-192. doi:10.1016/j.ajog.2019.10.096 31689381 PMC7261363

[12] Tarasoff LA, Ravindran S, Malik H, Salaeva D, Brown HK Maternal disability and risk for pregnancy, delivery, and postpartum complications: a systematic review and meta-analysis. Am J Obstet Gynecol. 2020;222(1):27.e1-27.e32. doi:10.1016/j.ajog.2019.07.015 31306650 PMC6937395

[13] Morton C, Le JT, Shahbandar L, Hammond C, Murphy EA, Kirschner KL Pregnancy outcomes of women with physical disabilities: a matched cohort study. PM R. 2013;5(2):90-98. doi:10.1016/j.pmrj.2012.10.011 23200116

[14] Schiff MA, Doody DR, Crane DA, Mueller BA Pregnancy outcomes among deaf women in Washington state, 1987-2012. Obstet Gynecol. 2017;130(5):953-960. doi:10.1097/AOG.0000000000002321 29016488 PMC5958610

[15] Brown HK, Cobigo V, Lunsky Y, Vigod SN Maternal and offspring outcomes in women with intellectual and developmental disabilities: a population-based cohort study. BJOG. 2017;124(5):757-765. doi:10.1111/1471-0528.14120 27222439

[16] Mitra M, Clements KM, Zhang J, Smith LD Disparities in adverse preconception risk factors between women with and without disabilities. Matern Child Health J. 2016;20(3):507-515. doi:10.1007/s10995-015-1848-1 26518009 PMC4754136

[17] Havercamp SM, Scandlin D, Roth M Health disparities among adults with developmental disabilities, adults with other disabilities, and adults not reporting disability in North Carolina. Public Health Rep. 2004;119(4):418-426. doi:10.1016/j.phr.2004.05.006 15219799 PMC1497651

[18] Parish SL, Rose RA, Andrews ME Income poverty and material hardship among US women with disabilities. Soc Serv Rev. 2009;83(1):33-52. doi:10.1086/598755

[19] Kim M, Kim H-J, Hong S, Fredriksen-Goldsen KI Health disparities among childrearing women with disabilities. Matern Child Health J. 2013;17(7):1260-1268. 22918712 10.1007/s10995-012-1118-4

[20] Wisdom JP, McGee MG, Horner-Johnson W, Michael YL, Adams E, Berlin M Health disparities between women with and without disabilities: a review of the research. Soc Work Public Health. 2010;25(3):368-386. doi:10.1080/19371910903240969 20446182 PMC3546827

[21] von Elm E, Altman DG, Egger M, Pocock SJ, Gøtzsche PC, Vandenbroucke JP; STROBE Initiative The Strengthening the Reporting of Observational Studies in Epidemiology (STROBE) statement: guidelines for reporting observational studies. Int J Surg. 2014;12(12):1495-1499. doi:10.1016/j.ijsu.2014.07.013 25046131

[22] Williams JI, Young WA Summary of studies on the quality of health care administrative databases in Canada In: Goel V, Williams JI, Anderson GM, Blackstien-Hirsch P, Fooks C, Naylor CD, eds. Patterns of Health Care in Ontario: The ICES Practice Atlas. 2nd Ed. Canadian Medical Association; 1996.

[23] Darney BG, Biel FM, Quigley BP, Caughey AB, Horner-Johnson W Primary cesarean delivery patterns among women with physical, sensory, or intellectual disabilities. Womens Health Issues. 2017;27(3):336-344. doi:10.1016/j.whi.2016.12.007 28109562 PMC5435518

[24] Lin E, Balogh R, Cobigo V, Ouellette-Kuntz H, Wilton AS, Lunsky Y Using administrative health data to identify individuals with intellectual and developmental disabilities: a comparison of algorithms. J Intellect Disabil Res. 2013;57(5):462-477. doi:10.1111/jir.12002 23116328

[25] Agency for Healthcare Research and Quality Beta Chronic Condition Indicator (CCI) for ICD-10-CM: Healthcare Cost and Utilization Project (HCUP). Agency for Healthcare Research & Quality; 2018.

[26] World Health Organization International Classification of Functioning, Disability and Health: ICF. World Health Organization; 2001.

[27] You JJ, Alter DA, Stukel TA, Proliferation of prenatal ultrasonography. CMAJ. 2010;182(2):143-151. doi:10.1503/cmaj.090979 20048009 PMC2817321

[28] Ray JG, Park AL, Dzakpasu S, Prevalence of severe maternal morbidity and factors associated with maternal mortality in Ontario, Canada. JAMA Netw Open. 2018;1(7):e184571. doi:10.1001/jamanetworkopen.2018.4571 30646359 PMC6324398

[29] Kralj B. Measuring “rurality” for purposes of health-care planning: an empirical measure for Ontario. Ont Med Rev. 2000;67(9):33-52.11874939

[30] Hux JE, Ivis F, Flintoft V, Bica A Diabetes in Ontario: determination of prevalence and incidence using a validated administrative data algorithm. Diabetes Care. 2002;25(3):512-516. doi:10.2337/diacare.25.3.512 11874939

[31] Tu K, Campbell NR, Chen Z-L, Cauch-Dudek KJ, McAlister FA Accuracy of administrative databases in identifying patients with hypertension. Open Med. 2007;1(1):e18-e26. 20101286 PMC2801913

[32] Tu K, Wang M, Young J, Validity of administrative data for identifying patients who have had a stroke or transient ischemic attack using EMRALD as a reference standard. Can J Cardiol. 2013;29(11):1388-1394. doi:10.1016/j.cjca.2013.07.676 24075778

[33] Tu K, Nieuwlaat R, Cheng SY, Identifying patients with atrial fibrillation in administrative data. Can J Cardiol. 2016;32(12):1561-1565. doi:10.1016/j.cjca.2016.06.006 27742459

[34] Schultz SE, Rothwell DM, Chen Z, Tu K Identifying cases of congestive heart failure from administrative data: a validation study using primary care patient records. Chronic Dis Inj Can. 2013;33(3):160-166. doi:10.24095/hpcdp.33.3.06 23735455

[35] Tu K, Mitiku T, Guo H, Lee DS, Tu JV Myocardial infarction and the validation of physician billing and hospitalization data using electronic medical records. Chronic Dis Can. 2010;30(4):141-146. doi:10.24095/hpcdp.30.4.06 20946715

[36] The Johns Hopkins University The Johns Hopkins ACG system: technical reference guide version 10.0, 2013. Accessed December 17, 2020. https://www.hopkinsacg.org/document/acg-system-version-10-0-technical-reference-guide/

[37] Kurdyak P, Lin E, Green D, Vigod S Validation of a population-based algorithm to detect chronic psychotic illness. Can J Psychiatry. 2015;60(8):362-368. doi:10.1177/070674371506000805 26454558 PMC4542516

[38] Steele LS, Glazier RH, Lin E, Evans M Using administrative data to measure ambulatory mental health service provision in primary care. Med Care. 2004;42(10):960-965. doi:10.1097/00005650-200410000-00004 15377928

[39] Austin PC Using the standardized difference to compare the prevalence of a binary variable between two groups in observational research. Commun Stat Simul Comput. 2009;38(6):1228-1234. doi:10.1080/03610910902859574

[40] Zou G A modified Poisson regression approach to prospective studies with binary data. Am J Epidemiol. 2004;159(7):702-706. doi:10.1093/aje/kwh090 15033648

[41] Zou GY, Donner A Extension of the modified Poisson regression model to prospective studies with correlated binary data. Stat Methods Med Res. 2013;22(6):661-670. doi:10.1177/0962280211427759 22072596

[42] Molina G, Weiser TG, Lipsitz SR, Relationship between cesarean delivery rate and maternal and neonatal mortality. JAMA. 2015;314(21):2263-2270. doi:10.1001/jama.2015.15553 26624825

[43] Wall-Wieler E, Carmichael SL, Gibbs RS, Severe maternal morbidity among stillbirth and live birth deliveries in California. Obstet Gynecol. 2019;134(2):310-317. doi:10.1097/AOG.0000000000003370 31306335 PMC6921936

[44] Kim MK, Lee SM, Bae S-H, Socioeconomic status can affect pregnancy outcomes and complications, even with a universal healthcare system. Int J Equity Health. 2018;17(1):2. doi:10.1186/s12939-017-0715-7 29304810 PMC5756361

[45] Andresen EM, Brownson RC Disability and health status: ethnic differences among women in the United States. J Epidemiol Community Health. 2000;54(3):200-206. doi:10.1136/jech.54.3.200 10746114 PMC1731647

[46] Louis JM, Menard MK, Gee RE Racial and ethnic disparities in maternal morbidity and mortality. Obstet Gynecol. 2015;125(3):690-694. doi:10.1097/AOG.0000000000000704 25730234

[47] Misra DP, Grason H, Weisman C An intersection of women’s and perinatal health: the role of chronic conditions. Womens Health Issues. 2000;10(5):256-267. doi:10.1016/S1049-3867(00)00054-2 10980443

[48] Iezzoni LI Using administrative data to study persons with disabilities. Milbank Q. 2002;80(2):347-379. doi:10.1111/1468-0009.t01-1-00007 12101876 PMC2690114

[49] Lamadrid-Figueroa H, Montoya A, Fritz J, Olvera M, Torres LM, Lozano R Towards an inclusive and evidence-based definition of the maternal mortality ratio: an analysis of the distribution of time after delivery of maternal deaths in Mexico, 2010-2013. PLoS One. 2016;11(6):e0157495. doi:10.1371/journal.pone.0157495 27310260 PMC4911006

[50] Crear-Perry J, Correa-de-araujo R, Lewis Johnson T, McLemore MR, Neilson E, Wallace M Social and structural determinants of health inequities in maternal health. J Womens Health (Larchmt). 2020. doi:10.1089/jwh.2020.8882 33181043 PMC8020519

