# Supplementary material for: Association of Preexisting Disability With Severe Maternal Morbidity or Mortality in Ontario, Canada
Source: JAMA Netw Open. 2021 Feb 8;4(2):e2034993. doi: 10.1001/jamanetworkopen.2020.34993 (PMC7871190; doi:10.1001/jamanetworkopen.2020.34993)
Supplement: Supplement. — eTable 1. Details of Health Administrative Data Sources eTable 2. Details of How a Physical, Sensory, or Intellectual/Developmental Disability Was Determined eTable 3. Details of How Severe Maternal Morbidity and Mortality Were Determined eTable 4. Risk of Severe Maternal Morbidity or Mortality Arising Between Conception and 365 Days Post Partum in Women With a Disability by (a) the Age at Which a Disability Was Diagnosed and (b) the Timing of a Disability-Related Health Care Encounter Prior to Conception Compared With Women Without a Disability eTable 5. Risk of Severe Maternal Morbidity or Mortality Arising Between Conception and 365 Days Post Partum in Women With a Disability, by Subtype of Disability, Compared With Women Without a Disability eTable 6. Risk of Severe Maternal Morbidity or Mortality, Arising Between Birth and 365 Days Post Partum in Women With a Disability, Compared With Women Without a Disability, Stratified by (a) Delivery Mode and (b) Birth Outcome [file jamanetwopen-e2034993-s001.pdf]

Supplemental Online Content

Brown HK, Ray JG, Chen S, et al. Association of preexisting disability with severe maternal morbidity or mortality in Ontario, Canada. *JAMA Netw Open*. 4(2):e2034993. doi:10.1001/jamanetworkopen.2020.34993

eTable 1. Details of Health Administrative Data Sources

eTable 2. Details of How a Physical, Sensory, or Intellectual/Developmental Disability Was Determined

eTable 3. Details of How Severe Maternal Morbidity and Mortality Were Determined

eTable 4. Risk of Severe Maternal Morbidity or Mortality Arising Between Conception and 365 Days Post Partum in Women With a Disability by (a) the Age at Which a Disability Was Diagnosed and (b) the Timing of a Disability-Related Health Care Encounter Prior to Conception Compared With Women Without a Disability

eTable 5. Risk of Severe Maternal Morbidity or Mortality Arising Between Conception and 365 Days Post Partum in Women With a Disability, by Subtype of Disability, Compared With Women Without a Disability

eTable 6. Risk of Severe Maternal Morbidity or Mortality, Arising Between Birth and 365 Days Post Partum in Women With a Disability, Compared With Women Without a Disability, Stratified by (a) Delivery Mode and (b) Birth Outcome

This supplemental material has been provided by the authors to give readers additional information about their work.

© 2021 Brown HK et al. *JAMA Network Open.*

eTable 1. Details of Health Administrative Data Sources

Data source	Construct	Coding structure	Inception
Canadian Institute for Health Information Discharge Abstract Database	Hospital admissions	Canadian Coding Standards for the International Classification of Diseases and Related Health Problems codes for diagnoses and Canadian Classification of Health Interventions codes for procedures	1988
National Ambulatory Care Reporting System	Emergency department visits	Canadian Coding Standards for the International Classification of Diseases and Related Health Problems codes for diagnoses and Canadian Classification of Health Interventions codes for procedures	2000
Office of the Registrar General Vital Statistics Death Registry	Deaths	International Classification of Diseases and Related Health Problems codes for cause of death	1990
Ontario Health Insurance Database	Outpatient physician visits	Physician billing codes	1991
Ontario Mental Health Reporting System	Psychiatric hospital admissions	Diagnostic and Statistical Manual of Mental Disorders	2005
Registered Persons Database	Sociodemographic data	N/A	1991

© 2021 Brown HK et al. *JAMA Network Open.*

eTable 2. Details of How a Physical, Sensory, or Intellectual/Developmental Disability Was Determined

Category	Condition name	ICD-10 codes	ICD-9 codes	Other codes
Physical disability:	* Congenital deformities of the spine (e.g., congenital scoliosis)	Q67.5	754.2	
Congenital anomalies	Congenital deformities of the feet (e.g., club foot)	Q66	754.5-754.7	OHIP: 754
	Congenital musculoskeletal deformities of the chest (e.g., congenital funnel chest)	Q67.6, Q67.7, Q67.8	754.8	
	Dwarfism, not elsewhere classified	E34.3	259.4	
	Hypopituitarism (e.g., pituitary dwarfism)	E23.0	253.4	
	Other congenital anomalies of the nervous system (e.g., congenital hydrocephalus)	Q01.9, Q02-Q04, Q06, Q07.8, Q07.9, G90.1	742	OHIP: 742
	Other congenital musculoskeletal deformities (e.g., osteochondroplasia)	Q75-Q79	756	OHIP: 756
	Reduction defects of lower limb	Q72	755.3	
	* Reduction defects of unspecified limb (e.g., phocomelia NOS)	Q73, Q74	755.4	
	Reduction defects of upper limb	Q71	755.2	
	Spina bifida	Q05	741	OHIP: 741
	Syndactyly	Q70	755.1	OHIP: 755
Physical disability: Musculoskeletal disorders	Acromegaly and gigantism	E22.0	253.0	
	Ankylosing spondylitis	M45, M46	720	OHIP: 720
	* Chronic osteomyelitis	M86.3-M86.6	730.1	
	Disc disorders	M50.0, M50.2-M50.9, M51.0, M51.2-M51.9	722	
	Internal derangement of the knee	M22.4, M23.2-M23.5, M23.8, M23.9	717	
	Osteoarthritis	M15-M19	715	OHIP: 715
	Osteochondropathies	M42, M91, M92, M93	732	OHIP: 732
	* Osteonecrosis	M87	733.4	
	* Osteoporosis with history of pathological fracture	M80	733.1	
	Polymyalgia rheumatica	M35.3	725	OHIP: 725
	Rheumatoid arthritis	M05, M06	714	OHIP: 714
	Spondylosis	M47	721	OHIP: 721
Physical disability: Neurological	Cerebral palsy	G80	343	OHIP: 343
	Disorders of autonomic nervous system (e.g., idiopathic peripheral autonomic neuropathy)	G90	337	

© 2021 Brown HK et al. *JAMA Network Open.*

Category	Condition name	ICD-10 codes	ICD-9 codes	Other codes
disorders	Epilepsy	G40	345.0-345.1, 345.4-345.9	OHIP: 345
	Hemiplegia	G81	342	
	Hereditary and idiopathic neuropathy	G60	356	OHIP: 356
	Hereditary ataxia and other specified degenerative disorders of the nervous system classified elsewhere (e.g., Huntington's disease)	G11, G32.8	334	
	* Mononeuropathies of the lower limb	G57, G58	355	
	Multiple sclerosis	G35	340	OHIP: 340
	Muscular dystrophy	G71, G72	359.0	OHIP: 359
	Myasthenia gravis	G70	358	OHIP: 358
	Nerve root and plexus disorders	G54, G55	353	
	Other demyelinating diseases of central nervous system (e.g., diffuse sclerosis)	G36, G37	341	
	Other disorders of spinal cord (e.g., syringomyelia)	G95	336	
	Other extrapyramidal and movement disorders (e.g., essential tremor)	G10, G23, G24.1-G24.9, G25	333	
	Other paralytic syndromes (e.g., paraplegia)	G82, G83	344	
	Other polyneuropathies (e.g., Guillain-Barre syndrome)	G61, G62, G63	357	
	Other specified degenerative diseases of the nervous system (e.g., cerebral ataxia)	G31.8	331.8	
	Parkinson's disease	G20, G21	332.0-332.1	OHIP: 332
	Sequelae of cardiovascular disease	I69	438	
	Sequelae of poliomyelitis	B91	138	
	Spinal muscular atrophy and related syndromes (e.g., ALS)	G12	335.1, 335.2, 335.8, 335.9	
Physical Disability: Permanent injuries	* Brain injury	S02.0, S02.1, S02.3, S02.7-S02.9, S06.1-S06.9, S07, T02.0, T90.5	800.1, 800.3, 801.1, 801.3, 802.6, 802.7, 803.1, 803.3, 804.1, 804.3, 850, 851-854, 907.0, 907.1	
	Crushing injury of the lower limb	S77, S87, S97.0, T04.1, T04.3-T04.8	928.0-928.2, 928.8	
	Dependence on a wheelchair	Z99.3	V46.3	
	Dependence on other enabling machines and devices	Z99.8	V46.8	

© 2021 Brown HK et al. *JAMA Network Open.*

Category	Condition name	ICD-10 codes	ICD-9 codes	Other codes
	* Fracture of the lower back or pelvis	S32.4-S32.8, T91.2	808	OHIP: 808
	Fracture of the vertebral column with spinal cord injury	S14.0, S14.1, S24.0, S24.1, S34.0, S34.1,	806, 907.2, 952	OHIP: 806
		S34.3, T06.0, T06.1, T91.3		
	Other acquired deformities of limbs	M21.8	736.8	
	Traumatic amputation of the lower limb	S78, S88, S98.0, S98.3, T05, Z89.4-Z89.8	896, 897, V49.7	
	Traumatic amputation of the upper limb	S48, S58, S68.3, S68.4, Z89.1-Z89.3	887, V49.6	
Sensory disabilities: Hearing impairments	Conductive and sensorineural hearing loss	H90, H91.3, H91.8, H91.9	389	389
	Congenital malformations of ear causing impairment of hearing	Q16.0, Q16.1, Q16.3-Q16.9	744.0	
Sensory disabilities: Vision impairments	Blindness and low vision	H54	369	369
	Cataracts	H25, H26	366	366
	Chorioretinal inflammation	H30, H31	363	
	* Congenital malformations of the eye	Q11.1, Q11.2, Q13.1, Q13.3, Q13.8, Q15.0	743.0-743.2, 743.4	
	Disorders of globe (e.g., hypotony of eye)	H44	360	
	Disorders of the iris and ciliary body (e.g., chronic iridocyclitis)	H20.1	364.1	
	Disorders of visual cortex	H47.6	377.7	
	Glaucoma	H40, H42	365	OHIP: 365
	* Nystagmus and other irregular eye movements	H55	379.5	
	Other retinal disorders (e.g., other proliferative retinopathy)	E10.31-E10.35, E11.31-E11.35, H34-H36	362	OHIP: 362
Intellectual and developmental disabilities	Autism spectrum disorder	F84.0, F84.1, F84.3-F84.9	299	OHIP: 299; OMHRS: Q2a, Q2b or Q2c (i.e., Axis I) in 299, 299.00, 299.1, 299.10, 299.8, 299.809 (and retired fields)

© 2021 Brown HK et al. *JAMA Network Open.*

Category	Condition name	ICD-10 codes	ICD-9 codes	Other codes
	Fetal alcohol spectrum disorder	Q86.0	760.71, 760.77 only if 5 digits exist	
	Intellectual disability	F70-F73, F78, F79	317-319	OHIP: 319; OMHRS: Q2d (i.e., Axis II) in 317, 318, 318.0, 318.1, 318.2, 319 (and retired fields)
	Intellectual disability resulting from chromosomal anomalies	Q90, Q91, Q92.0-Q92.5, Q92.7-Q92.9, Q93, Q97.1, Q99.2, Q99.8	758.0-758.3, 758.5, 758.8 (not 758.81, only if 5 digits exist), 758.9	OMHRS: I11h–I11m = any diagnosis of Qxxx as listed in ICD-10 column
	Other intellectual disabilities (e.g., fetal alcohol syndrome, tuberous sclerosis)	Q85.1, Q86.1, Q87.1, Q87.23, Q87.31, Q87.8	759.5, and the following only if 6 digits exist: 759.821 (not 759.82), 759.827, 759.828, 759.83, 759.874, 759.875, 759.89	OMHRS: I11h–I11m = any diagnosis of Qxxx as listed in ICD-10 column; Q3 = 1

Abbreviations: ICD = International Statistical Classification of Diseases and Related Health Problems; OHIP = Ontario Health Insurance Plan; OMHRS = Ontario Mental Health Reporting System.

* = added to the algorithms identified in our literature review.

© 2021 Brown HK et al. *JAMA Network Open.*

eTable 3. Details of How Severe Maternal Morbidity and Mortality Were Determined

Outcome	Codes or definition	Data source(s)
Acute abdomen	ICD-10: K35, K37, K65, N73.3, N73.5	CIHI-DAD
Acute fatty liver with red blood cell (RBC) or plasma transfusion	ICD-10: O26.6 + (RBCTRNSF='Y' or PLSTRNSF='Y')	CIHI-DAD
Acute psychosis	ICD-10: F53.1, F23	CIHI-DAD
Acute renal failure	ICD-10: O90.4, N17, N19, N99.0	CIHI-DAD
Adult respiratory distress syndrome	ICD-10: J80	CIHI-DAD
Antepartum hemorrhage with coagulation defect	ICD-10: O46.0	CIHI-DAD
Assisted ventilation through endotracheal tube	CCI: 1.GZ.31.CA-ND	CIHI-DAD
Assisted ventilation through tracheostomy	CCI: 1.GZ.31.CR-ND	CIHI-DAD
Cardiac conditions	ICD-10: O74.2, O75.4, O89.1, O90.3, I21, I22, I42, I43, I46, I49.0, I50, J81; CCI: 1.HZ.09, 1.HZ.30	CIHI-DAD
Cerebral edema or coma	ICD-10: G93.6, R40.2	CIHI-DAD
Cerebral venous thrombosis in pregnancy, or in the puerperium	ICD-10: O22.5 or O87.3	CIHI-DAD
Cerebrovascular diseases: subarachnoid and intracranial hemorrhage, cerebral infarction, stroke	ICD-10: I60-I64	CIHI-DAD
Complications of obstetric surgery and procedures	ICD-10: O75.4	CIHI-DAD
Curettage with RBC transfusion	(5.PC.91.GA, 5.PC.91.GC or 5.PC.91.GD) + RBCTRNSF='Y'	CIHI-DAD
Dialysis	CCI: 1.PZ.21^^	CIHI-DAD
Disseminated intravascular coagulation	ICD-10: D65	CIHI-DAD
Eclampsia	ICD-10: O15	CIHI-DAD
Evacuation of incisional hematoma with RBC transfusion	5.PC.73.JS + RBCTRNSF='Y'	CIHI-DAD
Hepatic failure	ICD-10: K71-K72	CIHI-DAD
Hysterectomy	CCI: 5.MD.60.RC, 5.MD.60.RD, 5.MD.60.KE, 5.MD.60.CB, 1.RM.89.LA (exclude if 1.PL.74, 1.RS.74 or 1.RS.80 code also present), 1.RM.87.LA-GX	CIHI-DAD
Intrapartum hemorrhage with coagulation defect	ICD-10: O67.0	CIHI-DAD
Intrapartum hemorrhage with RBC transfusion	ICD-10: O67 + blood transfusion	CIHI-DAD
Maternal ICU admission	FTSPCU in ('10', '20', '25', '30', '35', '40', '45', '60', '80')	CIHI-DAD
Maternal mortality	N/A	ORGD, RPDB
Obstetric embolism	ICD-10: O88	CIHI-DAD
Obstetric shock	ICD-10: O75.1, R57, T80.5, T88.6	CIHI-DAD

© 2021 Brown HK et al. *JAMA Network Open.*

Outcome	Codes or definition	Data source(s)
Placenta previa with hemorrhage with RBC transfusion	ICD-10: O44.1 + RBCTRNSF=‘Y’	CIHI-DAD
Placental abruption with coagulation defect	ICD-10: O45.0	CIHI-DAD
Postpartum hemorrhage with RBC transfusion, procedures to the uterus or hysterectomy	ICD-10: O72 + (RBCTRNSF=‘Y’ or [CCI: 1.RM.13^^ or 1.KT.51 or 5.PC.91.LA or 5.PC.91.HV + (RBCTRNSF=1)] or [CCI: 5.MD.60.RC, 5.MD.60.RD, 5.MD.60.KE, 5.MD.60.CB, 1.RM.89.LA] or 1.RM.87.LA-GX). Note: 1.RM.89.LA is included only if codes 1.PL.74, 1.RS.74 or 1.RS.80 are NOT also present	CIHI-DAD
Procedures to the uterus/pelvic vessels with RBC transfusion	(1.RM.13^^ or 1.KT.51 or 5.PC.91.LA) + blood transfusion	CIHI-DAD
Puerperal sepsis	ICD-10: O85	CIHI-DAD
Pulmonary, cardiac, and CNS complications of anaesthesia during pregnancy, the puerperium, or labour and delivery	ICD-10: O29.0, O29.1, O29.2, O74.0, O74.1, O74.2, O74.3, O89.0, O89.1, O89.2	CIHI-DAD
Reclosure of caesarean wound with RBC transfusion	(5.PC.80.JM or 5.PC.80.JH) + RBCTRNSF=‘Y’	CIHI-DAD
Repair of bladder, urethra, or intestine	CCI: 5.PC.80.JR, 1.NK.80^^, 1.NM.80^^	CIHI-DAD
Rupture of the uterus with RBC transfusion, procedures to the uterus or hysterectomy	(ICD-10: O71.0 or O71.1) + (RBCTRNSF=‘Y’ or [CCI: 1.RM.13^^ or 1.KT.51 or 5.PC.91.LAor 5.PC.91.HV] or [CCI: 5.MD.60.RC, 5.MD.60.RD, 5.MD.60.KE, 5.MD.60.CB, 1.RM.89.LA, 1.RM.87.LA-GX]). Note: 1.RM.89.LA is included only if codes 1.PL.74, 1.RS.74 or 1.RS.80 are NOT also present	CIHI-DAD
Septicemia during labour	ICD-10: O75.3	CIHI-DAD
Severe preeclampsia and HELLP syndrome	ICD-10: O14.1, O14.2	CIHI-DAD
Sickle cell anemia with crisis	ICD-10: D57.0	CIHI-DAD
Status asthmaticus	ICD-10: J45.01, J45.11, J45.81, J45.91	CIHI-DAD
Status epilepticus	ICD-10: G41	CIHI-DAD
Surgical or manual correction of inverted uterus for vaginal births only	CCI: 5.PC.91.HQ or 5.PC.91.HP, restricted to vaginal births (i.e., absence of caesarean code 5.MD.60)	CIHI-DAD

Abbreviations: CCI = Canadian Classification of Health Interventions; CIHI-DAD: Canadian Institute for Health Information Discharge Abstract Database; ICD = International Statistical Classification of Diseases and Related Health Problems; ORGD = Ontario Registrar General Vital Statistics Death Registry; RPDB = Registered Persons Database.

eTable 4. Risk of Severe Maternal Morbidity or Mortality Arising Between Conception and 365 Days Post Partum in Women With a Disability by (a) the Age at Which a Disability Was Diagnosed and (b) the Timing of a Disability-Related Health Care Encounter Prior to Conception Compared With Women Without a Disability

Variable	Disability type	Number (%) with outcome	Unadjusted RR (95% CI)	Model 1: Adjusted RR (95% CI)[a]	Model 2: Adjusted RR (95% CI)[b]
**Timing of disability diagnosis**[c]	None (N=731,238)	14,935 (2.0)	1.00 (Referent)	1.00 (Referent)	1.00 (Referent)
	Physical only	---	---	---	---
	< 15 years (N=38,178)	1,110 (2.9)	1.42 (1.33-1.51)	1.42 (1.33-1.51)	1.35 (1.26-1.43)
	≥ 15 years (N=44,611)	1,207 (2.7)	1.33 (1.25-1.41)	1.33 (1.25-1.41)	1.22 (1.15-1.30)
	Sensory only	---	---	---	---
	< 15 years (N=19,179)	431 (2.2)	1.10 (1.00-1.21)	1.10 (0.99-1.21)	1.07 (0.97-1.18)
	≥ 15 years (N=9,354)	249 (2.7)	1.30 (1.15-1.48)	1.29 (1.14-1.47)	1.17 (1.03-1.33)
	Intellectual/developmental only	---	---	---	---
	< 15 years (N=1,050)	45 (4.3)	2.00 (1.46-2.74)	1.96 (1.43-2.69)	1.81 (1.32-2.48)
	≥ 15 years (N=622)	28 (4.5)	2.26 (1.55-3.31)	2.27 (1.56-3.31)	1.88 (1.29-2.74)
	Multiple	---	---	---	---
	< 15 years (N=4,509)	188 (4.2)	2.08 (1.80-2.42)	2.07 (1.78-2.40)	1.77 (1.53-2.04)
	≥ 15 years (N=1,480)	52 (3.5)	1.72 (1.29-2.29)	1.69 (1.27-2.25)	1.30 (0.98-1.73)
**Proximity of disability-related health care use to conception**	None (N=1,601,363)	31,752 (2.0)	1.00 (Referent)	1.00 (Referent)	1.00 (Referent)
	Physical only	---	---	---	---
	< 1 year (N=26,714)	875 (3.3)	1.63 (1.52-1.75)	1.60 (1.49-1.71)	1.41 (1.32-1.51)
	≥ 1 years only (N=118,258)	3,237 (2.7)	1.38 (1.33-1.43)	1.37 (1.32-1.42)	1.29 (1.25-1.34)
	Sensory only	---	---	---	---
	< 1 year (N=7,463)	195 (2.6)	1.28 (1.11-1.48)	1.24 (1.08-1.43)	1.12 (0.97-1.29)
	≥ 1 years only (N=37,786)	904 (2.4)	1.21 (1.13-1.30)	1.20 (1.12-1.29)	1.15 (1.08-1.23)
	Intellectual/developmental only	---	---	---	---
	< 1 year (N=403)	18 (4.5)	2.16 (1.34-3.47)	2.10 (1.31-3.37)	1.70 (1.07-2.70)
	≥ 1 years only (N=1,824)	68 (3.7)	1.83 (1.42-2.36)	1.83 (1.42-2.35)	1.67 (1.30-2.15)
	Multiple	---	---	---	---
	< 1 year (N=2,788)	150 (5.4)	2.73 (2.31-3.23)	2.63 (2.22-3.11)	2.02 (1.71-2.38)
	≥ 1 years only (N=6,095)	224 (3.7)	1.85 (1.62-2.12)	1.83 (1.60-2.09)	1.61 (1.41-1.84)

[a] Adjusted for maternal age, parity, neighbourhood income quintile, and region of residence.
[b] Adjusted for maternal age, parity, neighbourhood income quintile, region of residence, type 1 or type 2 diabetes mellitus, chronic hypertension or cardiovascular disease, stable and unstable chronic conditions, mental illness, and substance use disorders.
[c] This analysis is restricted to women with at least 2 years of health administrative data prior to 15 years of age.

© 2021 Brown HK et al. *JAMA Network Open.*

eTable 5. Risk of Severe Maternal Morbidity or Mortality Arising Between Conception and 365 Days Post Partum in Women With a Disability, by Subtype of Disability, Compared With Women Without a Disability

Disability type	Number (%) with outcome	Unadjusted RR (95% CI)	Model 1: Adjusted RR (95% CI)[a]	Model 2: Adjusted RR (95% CI)[b]
None (N=1,601,363)	31,752 (2.0)	1.00 (Referent)	1.00 (Referent)	1.00 (Referent)
Physical only	---	---	---	---
Musculoskeletal only (N=66,343)	1,700 (2.6)	1.30 (1.24-1.36)	1.28 (1.22-1.34)	1.19 (1.13-1.25)
Neurological only (N=35,615)	1,121 (3.2)	1.57 (1.48-1.67)	1.56 (1.47-1.66)	1.43 (1.35-1.53)
Permanent injury only (N=22,202)	534 (2.4)	1.19 (1.09-1.30)	1.20 (1.10-1.31)	1.16 (1.06-1.27)
Congenital anomalies only (N=10,174)	321 (3.2)	1.57 (1.40-1.76)	1.59 (1.42-1.78)	1.53 (1.37-1.72)
Multiple physical (N=10,638)	436 (4.1)	2.08 (1.89-2.29)	2.05 (1.86-2.26)	1.79 (1.62-1.97)
Sensory only	---	---	---	---
Hearing loss only (N=32,554)	734 (2.3)	1.14 (1.06-1.23)	1.14 (1.06-1.23)	1.10 (1.03-1.19)
Vision loss only (N=12,282)	356 (2.9)	1.43 (1.29-1.60)	1.38 (1.24-1.54)	1.25 (1.12-1.39)
Multiple sensory (N=413)	9 (2.3)	1.13 (0.59-2.17)	1.09 (0.57-2.08)	0.96 (0.50-1.84)
Intellectual/developmental only (N=2,227)	86 (3.9)	1.89 (1.51-2.36)	1.89 (1.51-2.36)	1.67 (1.34-2.09)
Multiple (N=8,883)	---	---	---	---
Physical and sensory (N=7,902)	333 (4.2)	2.13 (1.91-2.38)	2.08 (1.86-2.33)	1.77 (1.58-1.97)
Physical or sensory and intellectual/ developmental (N=868)[c]	31 (3.6)	1.87 (1.30-2.69)	1.87 (1.30-2.68)	1.47 (1.03-2.09)
All three types (N=113)	10 (8.9)	4.49 (2.26-8.90)	4.45 (2.25-8.82)	3.10 (1.60-6.01)

[a] Adjusted for maternal age, parity, neighbourhood income quintile, and region of residence.

[b] Adjusted for maternal age, parity, neighbourhood income quintile, region of residence, type 1 or type 2 diabetes mellitus, chronic hypertension or cardiovascular disease, stable and unstable chronic conditions, mental illness, and substance use disorders.

[c] Groups combined due to small numbers of outcome events in women with physical and intellectual/developmental, or sensory and intellectual/developmental disabilities separately.

© 2021 Brown HK et al. *JAMA Network Open.*

eTable 6. Risk of Severe Maternal Morbidity or Mortality, Arising Between Birth and 365 Days Post Partum in Women With a Disability, Compared With Women Without a Disability, Stratified by (a) Delivery Mode and (b) Birth Outcome

Variable	Disability type	Number (%) with outcome	Unadjusted RR (95% CI)	Model 1: Adjusted RR (95% CI)[a]	Model 2: Adjusted RR (95% CI)[b]
Vaginal birth	None (N=1,164,165)	11,599 (1.0)	1.00 (Referent)	1.00 (Referent)	1.00 (Referent)
	Physical only (N=100,846)	1,390 (1.4)	1.37 (1.30-1.45)	1.36 (1.29-1.44)	1.28 (1.21-1.36)
	Sensory only (N=32,062)	350 (1.1)	1.10 (0.98-1.22)	1.08 (0.97-1.21)	1.05 (0.94-1.17)
	Intellectual/developmental only (N=1,628)	33 (2.0)	2.02 (1.41-2.90)	1.94 (1.36-2.79)	1.71 (1.19-2.44)
	Multiple (N=5,878)	115 (2.0)	1.97 (1.63-2.37)	1.92 (1.60-2.32)	1.69 (1.41-2.04)
Caesarean birth	None (N=437,198)	10,289 (2.4)	1.00 (Referent)	1.00 (Referent)	1.00 (Referent)
	Physical only (N=44,126)	1,388 (3.1)	1.34 (1.27-1.42)	1.34 (1.26-1.41)	1.26 (1.19-1.34)
	Sensory only (N=13,187)	382 (2.9)	1.23 (1.11-1.36)	1.22 (1.10-1.35)	1.16 (1.05-1.28)
	Intellectual/developmental only (N=599)	26 (4.3)	1.82 (1.24-2.66)	1.78 (1.22-2.62)	1.63 (1.11-2.39)
	Multiple (N=3,005)	118 (3.9)	1.68 (1.40-2.01)	1.65 (1.37-1.98)	1.42 (1.18-1.71)
Livebirth	None (N=1,593,354)	21,275 (1.3)	1.00 (Referent)	1.00 (Referent)	1.00 (Referent)
	Physical only (N=144,187)	2,699 (1.9)	1.40 (1.34-1.46)	1.39 (1.33-1.44)	1.30 (1.25-1.35)
	Sensory only (N=44,988)	714 (1.6)	1.19 (1.10-1.28)	1.18 (1.09-1.27)	1.12 (1.04-1.21)
	Intellectual/developmental only (N=2,207)	[c]	[c]	[c]	[c]
	Multiple (N=8,823)	223 (2.5)	1.89 (1.66-2.16)	1.86 (1.62-2.12)	1.59 (1.40-1.82)
Stillbirth	None (N=8,009)	613 (7.7)	1.00 (Referent)	1.00 (Referent)	1.00 (Referent)
	Physical only (N=785)	79 (10.1)	1.32 (1.05-1.66)	1.32 (1.05-1.66)	1.23 (0.98-1.54)
	Sensory only (N=261)	18 (6.9)	0.96 (0.61-1.51)	0.97 (0.62-1.53)	0.93 (0.60-1.45)
	Intellectual/developmental only (N=20)	[c]	[c]	[c]	[c]
	Multiple (N=60)	10 (16.7)	2.24 (1.23-4.10)	2.17 (1.19-3.98)	1.69 (0.95-3.01)

[a] Adjusted for maternal age, parity, neighbourhood income quintile, and region of residence.

[b] Adjusted for maternal age, parity, neighbourhood income quintile, region of residence, type 1 or type 2 diabetes mellitus, chronic hypertension or cardiovascular disease, stable and unstable chronic conditions, mental illness, and substance use disorders.

[c] Data are suppressed to protect patient privacy because the outcome was reported in < 6 patients.

© 2021 Brown HK et al. *JAMA Network Open.*
